# Changes in the Orexin System in Rats Exhibiting Learned Helplessness Behaviors

**DOI:** 10.3390/brainsci11121634

**Published:** 2021-12-10

**Authors:** Chung-Wei Hsu, Sabrina Wang

**Affiliations:** Institute of Anatomy and Cell Biology, School of Medicine, National Yang Ming Chiao Tung University, 155, Section 2, Li-Nung Street, Beitou District, Taipei 112, Taiwan; Feirc123@hotmail.com

**Keywords:** orexin, major depression, stress, anxiety, corticosterone

## Abstract

Orexin-A (OX-A) and orexin-B (OX-B) are neuropeptides produced in the hypothalamus. Preclinical and clinical studies suggest that depression and anxiety are associated with the orexin system. In the current study, we used the learned helplessness (LH) animal model of depression to identify rats displaying LH behaviors (LH rats) and those that did not (No-LH rats). We compared the number of orexin-containing neurons in the hypothalamus of LH, No-LH, and control rats. Orexin peptides, orexin receptor 1 (OXR1) and 2 (OXR2) in brain areas involved in major depression and serum OX-A and corticosterone (CORT) concentrations were quantified and compared between rat groups. We found that LH and No-LH rats displayed higher serum OX-A concentrations compared with control rats. Comparison between LH and No-LH rats revealed that No-LH rats had significantly higher OX-A levels in the brain, more OX-A neurons, and more OX-A neuron activation. LH rats had more OX-B neurons and more OX-B neuron activation. Orexin peptides and receptors in the brain areas involved in major depression exhibited different patterns in LH and NoLH rats. Our findings revealed that activation of OX-A neurons could promote resilient behaviors under stressful situations and OX-A and OX-B neuropeptides exhibit dissimilar functions in LH behaviors.

## 1. Introduction

### 1.1. Orexin System and Major Depression

Orexins (also known as hypocretins) include orexin A (OX-A) and orexin B (OX-B) neuropeptides produced by a population of neurons located in the hypothalamus that encompasses the lateral hypothalamus, dorsomedial hypothalamus, and perifornical hypothalamus. This small population of orexin-producing neurons has extensive projections throughout the central nervous system, including the brain regions implicated in psychiatric diseases, namely the prefrontal cortex, hippocampus, thalamus, dorsal raphe, ventral tegmental area, amygdala, and locus coeruleus [1]. Orexin peptides act on two differentially distributed G-protein–coupled receptors, OXR1 and OXR2, throughout the brain [2,3]. Since their discovery in 1998, these neuropeptides have been linked with several physiological functions, including appetite and sleep [4,5,6,7]. Orexins also play a role in positively regulating the cognitive processes of attention and memory and indirectly stimulating corticotropin-releasing hormone (CRH)-synthesizing neurons in the paraventricular nucleus of the hypothalamus [8,9,10]. The first hint of a potential link between the orexin system and major depression was derived from the clinical observation that the oscillating amplitude of the OX-A concentration in the cerebrospinal fluid (CSF) during the diurnal cycle was significantly reduced in patients with major depression compared with that in healthy controls, but the mean OX-A level tended to be higher in patients with depression than in controls [11]. In other studies, the OX-A level in CSF was negatively correlated with lassitude and slowness of movement symptoms and the ratings of global illness in depressive patients who have attempted suicide, and the CSF OX-A level was significantly lower in patients with major depressive disorder than in patients with adjustment disorder and dysthymia [12,13]. An orexin genotype study of patients with depression revealed that a polymorphism (Ile408Val) in the OXR1 gene *HCRTR1* significantly differed between cases and controls [14]. However, other studies have failed to observe a significant difference in CSF OX-A levels between patients with depression and healthy controls [15,16]. Overall, despite clinical studies indicating considerable variation, sufficient evidence exists to suggest that dysregulation of the orexin system could play a role in depressive disorder.

### 1.2. Changes in Orexin System in Animal Models of Major Depression

Preclinical studies using various animal models of depression to examine the roles of the orexin system in depression have reported conflicting results. Studies using Wistar–Kyoto (WKY) rats, a genetic animal model of depression, have uncovered reduced prepro-orexin mRNA expression, a lower OX-A level in various brain areas, and an 18% reduction in orexin neuron numbers, along with a 15% reduction in soma size, compared with control Wistar rats [17,18]. However, another genetic animal model of depression, Flinder Sensitive Line (FSL) rats, displayed increased immobility in the forced swim test (FST) but had a higher number of orexin-producing neurons in the hypothalamus [19]. Inconsistent results have also been obtained in animal models in which stress was used to induce depression-like behaviors. Direct intracerebroventricular injection of OX-A in mice exhibited an antidepressant-like effect by decreasing immobility in the FST, whereas systemic dual-orexin receptor blockade also exerted antidepressant effects in the tail suspension test [20,21]. Genetic studies using OXR1 knockout mice have also demonstrated a decrease in depression-like behavior in the FST and tail suspension test, whereas OXR2 knockout animals displayed increased behavioral despair [22]. Evidence suggests that the signaling of these two receptors differentially regulates depression-like behaviors [22,23,24].

### 1.3. Learned Helplessness Animal Model of Depression

The LH animal model of depression is based on cognitive behavioral theory [25], which suggests that helplessness and hopelessness are potential etiologic factors that precede the onset of depression. To establish this model, animals are administered uncontrollable and inescapable foot or tail shocks [26]. Later, when avoidance tests (ATs) are administered, some animals display LH behavior in that they accept shocks even when they have opportunities to avoid them. This LH animal model has been used in preclinical pharmacologic studies to screen for the antidepressant effects of new compounds, and all major antidepressant compounds currently used, including tricyclic antidepressants, monoamine oxidase inhibitors [27], atypical antidepressants [28,29], and specific serotonergic reuptake inhibitors [28], have been shown to prevent and reverse LH in animals. Thus, this animal model of depression is considered to have high validity [30]. In order to further delineate the roles of the orexin system in major depression, we intend to use the LH model of depression to investigate changes in orexin and receptors in multiple brain areas involved in major depression. In the present study, we examined orexin peptides and two orexin receptors, OXR1 and OXR2, in behaviorally distinct LH and No-LH rats in comparison with controls. We observed differences in the orexin system between LH and No-LH rats. The results will help elucidate the roles of the orexin system in the stress response and depression-like behaviors.

## 2. Materials and Methods

### 2.1. Animals

Male Sprague–Dawley rats (BioLASCO Taiwan Co., Ltd., Taipei, Taiwan) 6–8 weeks old were used in this study. Upon arrival, rats were housed individually at the Laboratory Animals Center of National Yang-Ming University under a controlled environment with an ambient temperature of 24 °C and a 12-h light/dark cycle with the light on from 6:00 a.m. to 6:00 p.m. Food and water were provided ad libitum. Because rats were housed separately, toys were provided to prevent boredom and reduce isolation stress. After 1 week of acclimation, behavioral experiments were initiated. All procedures involving animal care and handling were preapproved by the Institutional Animal Care and Use Committee of National Yang-Ming University.

### 2.2. LH Protocol

**Figure 1:**
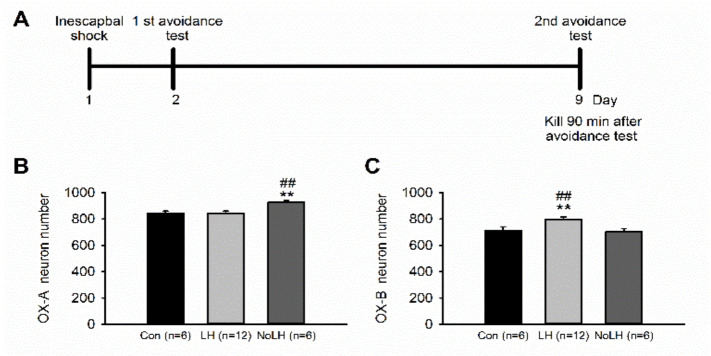
Timeline of experiments and the number of hypothalamic OX-A– and OX-B–positive neurons in LH, No-LH, and control rats. (**A**) Experimental timeline. After two ATs, rats were sacrificed 90 min after the second AT to record the peak c-fos expression window. (**B**) The number of OX-A neurons was significantly higher in the No-LH group (one-way ANOVA, between groups, *p* = 0.011; post hoc Bonferroni test, No-LH vs. Con, *p* = 0.039, No-LH vs. LH, *p* = 0.013) (**C**) The number of OX-B neurons was significantly higher in the LH group (one-way ANOVA, between groups, *p* = 0.01; post hoc Bonferroni test, No-LH vs. Con, *p* = 0.047, No-LH vs. LH, *p* = 0.026) ** *p* < 0.01, compared with Con; ## *p* < 0.01, comparison between LH and No-LH rats.

The LH protocol was conducted between 7:00 p.m. and 10:00 p.m. during the dark phase of the light/dark cycle. In the LH protocol, an animal was exposed to an inescapable shock (IS) on one side of a shuttle box. The IS consisted of 60 foot shocks of 0.4 mA accompanied by a tone. A 15-s shock was delivered every 5–50 s, with a random intershock interval, and the total time in the shuttle box was 60 min. Each animal in the control group (*n* = 12) was placed in the shuttle box for 60 min with no shock delivered. On day 2, each experimental animal underwent the AT as a postshock test, in which the animal could escape from the shock through an open gate dividing the two compartments of the shuttle box. The AT consisted of 15 trials. Animals were presented with a 3-s tone followed by an additional 10-s tone plus shock (0.4 mA, intertrial interval: 25–35 s). The gate between the two compartments of the shuttle box was opened at the onset of the tone. When the rat crossed into the other side (safe side) of the box, the gate closed, and the tone and shock terminated. If the rat failed to escape, the shock was terminated at the end of the trial, and the gate was closed. Rats with more than five escape failures in the last 10 trials were considered to have met the LH criterion. Control rats were placed in the shuttle box for 20 min on day 2 without shock treatment. On day 9, IS rats received a second AT, and control rats were placed in the shuttle box for 20 min without shock as on day 2. Based on the previous escape failure criteria, rats were assigned to the LH group (*n* = 21) or the No-LH group (*n* = 16); only rats that failed to escape in both ATs were assigned to the LH group. The AT shock times of LH and No-LH rats are presented in Supplementary Figure S1. The experimental timeline is illustrated in Figure 1A.

### 2.3. Brain Sample Collection

Ninety min after the second AT, rats were anesthetized through isoflurane (4%) inhalation, and a blood sample was collected from the left ventricle, after which brief ice-cold phosphate-buffered saline (PBS) perfusion was performed. For Western blotting, brain samples experiments were immediately harvested and quickly frozen in −80 °C 2-methylbutane for 10–15 s before being packaged in aluminum foil and stored at −80 °C until use. For collecting brain samples for immunohistochemical staining experiments, the rat brain was perfused with ice-cold PBS perfusion and then 4% paraformaldehyde for 4–6 min. The brain was then removed and fixed in 4% paraformaldehyde overnight. On the next day, the brain was removed from paraformaldehyde and stored in 0.3% sodium azide in PBS until sectioning.

### 2.4. Immunohistochemical Experiments

The stored brain samples were washed thrice in PBS for 5 min. Thereafter, the samples were placed in the cryoprotectant of sucrose (30% sucrose in 0.1 M PBS, pH 7.4) for 48 h. The sucrose cryoprotectant was replaced with fresh solution daily until the brain sank to the bottom of the container. The brain tissues were then washed in PBS thrice, embedded in Cryo-gel, and frozen in a −20 °C freezer. The frozen tissues were cut into 30-μm sections with a cryostat microtome. Sections were first washed in PBS to remove embedding gel and then stored in 0.3% sodium azide in PBS until use.

Brain sections containing the hypothalamus were used. We selected sections starting approximately −2.46 mm of the bregma where orexin neurons began to appear in the lateral hypothalamic area and the perifornical area. We randomly selected four sections from rostral to caudal, and each section was approximately 180 μm apart. The selected sections were washed thrice in PBS and then placed in blocking solution (10% bovine serum albumin mixed in 0.5% triton-X 100 in PBS) for 1 h. After blocking, the sections were incubated with primary antibodies in 0.5% triton-X 100 overnight at 4 °C. The primary antibodies and concentrations used were as follows: rabbit anti-OX-A antibody (1:1500, AB3704, Millipore, Chemicon International, Inc., Billerica, MA, USA), rabbit anti-OX-B antibody (1:500, GTX10982, GeneTex, Inc., Irvine, CA, USA), and mouse anti-c-Fos antibody (1:500, AB209842, Abcam, Cambridge, UK). On the second day, the sections were washed thrice to remove primary antibodies and then incubated with appropriate secondary antibodies at room temperature for 2 h. For fluorescent double staining, the secondary antibodies used were chicken anti-rabbit Alexa Fluor 594 (1:200, A-21442, Thermo Fisher Scientific, Waltham, MA, USA) and goat anti-mouse Alexa Fluor 488 (1:200, A-11001, Thermo Fisher Scientific, Waltham, MA, USA). For 3,3ʹ-diaminobenzidine (DAB) staining, the secondary antibodies used were biotinylated anti-rabbit IgG made in goat and streptavidin-conjugated horseradish peroxidase (HRP) (VECTASTAIN Elite ABC Kit, Vector Laboratories, Inc. Burlingame, CA, USA). We used a high-sensitivity substrate–chromogen system to detect reaction signals (Dako, Agilent Technologies Company, Glostrup, Denmark). Immunopositive cells were counted using an optical dissector method under a Leica DM6000B microscope (Leica, Mannheim, Germany) [31]. Double-labeled cells were counted under an Olympus FV1000 confocal microscope (Olympus corp., Tokyo, Japan).

### 2.5. Western Blotting

Whole-brain samples were placed in an ice-cold tissue blocker and cut into 1-mm-thick sections with razor blades. Using the rat brain atlas [32] for reference, we collected samples from the medial prefrontal cortex (mPFC, 3.7 to 2.2 mm of the bregma), nucleus accumbens (NAc, 1.7 to 0.2 mm of the bregma), and ventral tegmental area (VTA, −4.8 to −5.3 mm of the bregma). From sections −2.3 to −3.8 mm of the bregma, we collected the dentate gyrus (DG) of the hippocampal formation, the hypothalamus (Hyp), and the amygdala (Amy) by using a sample puncher collection needle (Harris Uni-Core, 0.75–1.2 mm; see Supplementary Figure S2 for punch locations). The collected samples were placed in protein extraction reagent buffer (Thermo Fisher Scientific, Inc. Waltham, MA, USA) containing a protease inhibitor. The samples were homogenized on a Minilys homogenizer (Precellys Homogenizers, Bertin Inc., Montigny-le-Bretonneux, France) with ceramic beads, shaking at 5000 rpm for 25 s. The homogenized solutions were collected in clean Eppendorf tubes and centrifuged at 16,392 RCF for 10 min at 4 °C. The supernatants were collected and stored at −80 °C until use. The protein concentration of each sample was quantified through the Bradford protein assay using BSA as a standard solution. The samples were denatured by heating at 95 °C for 5 min, and proteins were then separated through 8% or 15% SDS polyacrylamide gel electrophoresis and transferred to a polyvinylidene fluoride (PVDF) membrane (Pall, Inc. New York, NY, USA) The protein molecular weight stander used were PageRuler prestained protein ladder (26616, Thermo Fisher Scientific Inc. Waltham, MA, USA). The membrane was blocked with blocking buffer (10% fish gelatin in TBS-Tween 20) on a shaker at room temperature for 60 min and was then incubated with primary antibodies for 12–16 h at 4 °C. The primary antibodies used were rabbit anti-OX-A (AB3704), rabbit anti-OX-B (AB3100), rabbit anti-OXR1 (AB3092), or rabbit anti-OXR2 (AB3094) at 1:1000 concentrations (Millipore, Chemicon International, Inc., Temecula, CA, USA). The cadherin (1:10,000, GTX26528) and β-actin (1:30,000, GTX109639) antibodies were supplied by GeneTex, Inc (Irvine, CA, USA). The membranes were then washed thrice in TBST for 10 min, followed by the addition of secondary antibodies (anti-rabbit IgG conjugated with HRP, 1:5000, AB_2307391, Jackson Immuno Research, Inc., West Grove, PA, USA) and shaking for 2 h at room temperature. The target proteins were detected using ECL plus detection solution (PerkinElmer, Inc. Waltham, MA, USA) and were imaged using a luminescence imaging system (LAS-4000, GE healthcare, Wauwatosa, WI, USA). Finally, the membranes were incubated for 30 min in stripping buffer (62.6 mM Tris-HCl, 2% SDS, 100 mM β-mercaptoethanol, pH 6.8) and reprobed with anti-β-actin or anti-cadherin antibody (GeneTex, Inc., Irvine, CA, USA) as an internal control. The density of each band was quantified using ImageJ software (National Institutes of Health, Bethesda, MD, USA).

### 2.6. Enzyme-Linked Immunosorbent Assay

We used commercial enzyme-linked immunosorbent assay (ELISA) kits to measure the OX-A (USCNCEA607Ra, Wuhan USCN Business Co., Ltd., Wuhan, China), OX-B (M:BS267360, MyBioSource, Inc., San Diego, CA, USA), and CORT (ADI-900-097, Enzo Life Science Inc., Farmingdale, NY, USA) concentrations in the rat serum samples. The procedures were conducted following manufacturer instructions.

### 2.7. Statistics

For comparisons between the two groups, we used Student’s *t*-test. For multiple treatment group comparisons, we used a one-way analysis of variance (one-way ANOVA). Differences between the groups were determined through post hoc Bonferroni pairwise multiple comparison analysis. The software used was the statistical analysis section of SigmaPlot V.12 (Systat Software Inc., San Jose, CA, USA). The significance level in all analyses was 0.05. Values in the text and figures are expressed as mean ± standard error of mean (SEM).

## 3. Results

### 3.1. Orexin Neuronal Changes in LH and No-LH Rats

**Figure 2:**
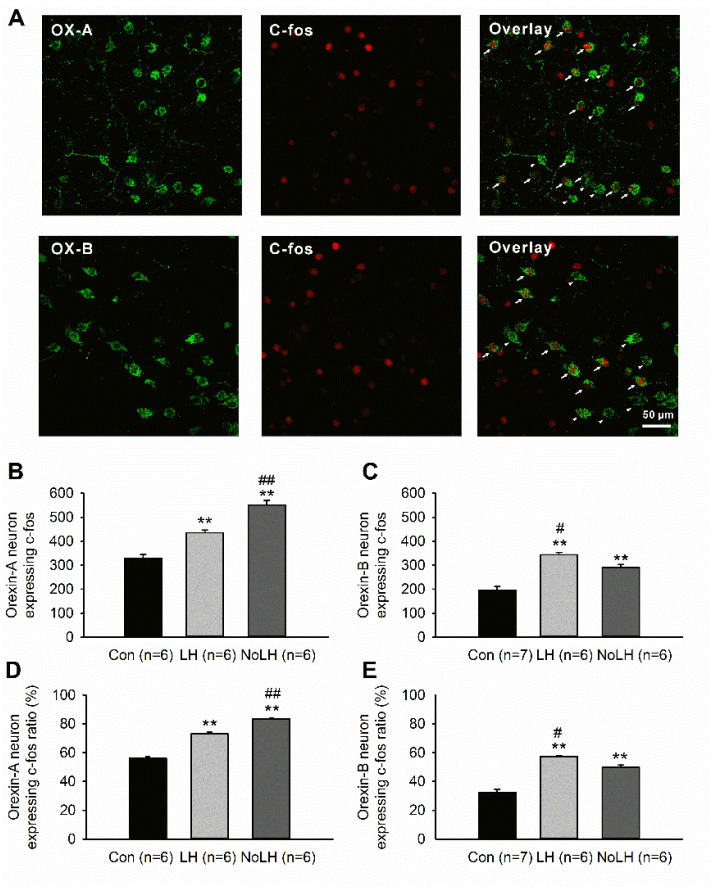
Activation of hypothalamic OX-A and OX-B neurons after the avoidance test. (**A**) Example of confocal images of c-fos and OX-A/OX-B in double-stained section. Arrowheads indicate OX-A or OX-B cells without c-fos, and arrows indicate double-labeled cells. (**B**) Number of OX-A neurons coexpressing c-fos. Both LH and No-LH rats exhibited significantly more OX-A activation than controls, and No-LH rats also showed more activation than LH rats (one-way ANOVA, between groups, *p* < 0.001; post hoc Bonferroni test, LH vs. Con, *p* < 0.001, No-LH vs. Con, *p* < 0.001, No-LH vs. LH, *p* < 0.001). (**C**) Number of OX-B neurons coexpressing c-fos. Both LH and No-LH rats displayed significantly more OX-B activation than controls, and LH rats also had more activation than No-LH rats (one-way ANOVA, between groups, *p* < 0.001; post hoc Bonferroni test, LH vs. Con, *p* < 0.001, No-LH vs. Con, *p* < 0.001, LH vs. No-LH, *p* = 0.034). (**D**) Ratio of OX-A neurons expressing c-fos. LH and No-LH rats had higher ratios than controls, and those of No-LH rats were significantly higher than those of LH rats (one-way ANOVA, between groups, *p* < 0.001; post hoc Bonferroni test, LH vs. Con, *p* < 0.001, No-LH vs. Con, *p* < 0.001, No-LH vs. LH, *p* < 0.001). (**E**) Ratio of OX-B neurons expressing c-fos. LH and No-LH rats exhibited higher ratios than controls, and those of LH rats were significantly higher than those of No-LH rats (one-way ANOVA, between groups, *p* < 0.001; post hoc Bonferroni test, LH vs. Con, *p* < 0.001, No-LH vs. Con, *p* < 0.001, No-LH vs. LH, *p* = 0.024). ** *p* < 0.01, compared with Con; # *p* < 0.05, ## *p* < 0.01, comparison between LH and No-LH rats.

We used the LH paradigm to identify rats that exhibited LH behaviors (LH rats) and those that did not (No-LH rats). Among the 37 tested rats, 21 were LH rats, and 16 were No-LH rats. In No-LH rats, the number of hypothalamic OX-A–positive neurons was significantly higher than that in control and LH rats (Figure 1B). By contrast, the number of OX-B–positive neurons was significantly higher in LH rats than in No-LH and control rats (Figure 1C). We also performed OX-A or OX-B and c-fos double-staining experiments to examine the activation pattern of these orexin neurons in LH, No-LH, and control rats. Regarding OX-A–positive neurons, both LH and No-LH rats exhibited significantly more activated OX-A–positive neurons than control rats (Figure 2A). In addition, the number of activated OX-A–positive neurons in No-LH rats was significantly higher than that in LH rats (Figure 2A). Regarding OX-B–positive neurons, both LH and No-LH rats also had more activated OX-B–positive neurons than control rats, with LH rats displaying significantly more activated OX-B–positive neurons than No-LH rats (Figure 2C). The same pattern was found for the ratio of activated OX-A and OX-B neurons, corresponding to the activated cell number (Figure 2D,E). These results indicated that rats that did not display helplessness behaviors had more OX-A–positive neuron activation in the hypothalamus, whereas rats that displayed helplessness behaviors had greater OX-B–positive neuron activation.

### 3.2. OX-A and CORT Concentrations in Serum

**Figure 3:**
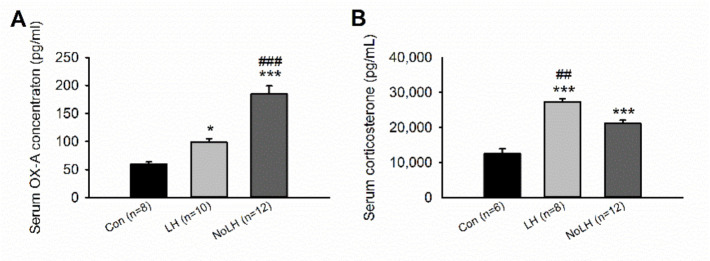
Serum OX-A peptide and corticosterone concentrations of LH, No-LH, and Con rats. (**A**) Both LH and No-LH rats had higher serum OX-A peptide concentrations than controls; the serum OX-A concentration of No-LH rats was also significantly higher than that of LH rats (one-way ANOVA, between groups, *p* < 0.001; post hoc Bonferroni test, LH vs. Con, *p* = 0.037, No-LH vs. Con, *p* < 0.001, No-LH vs. LH, *p* < 0.001). (**B**) Both LH and No-LH rats had higher serum corticosterone concentration than controls, and the corticosterone concentration of LH rats was significantly higher than that of No-LH rats (one-way ANOVA, between groups, *p* <  0.001; post hoc Bonferroni test, LH vs. Con, *p* < 0.001, No-LH vs. Con, *p* < 0.001, No-LH vs. LH, *p* < 0.001). * *p* < 0.05, *** *p* < 0.001, compared with Con; ## *p* < 0.01, ### *p* < 0.001, comparison between LH and No-LH rats.

Studies have indicated that OX-A can pass through the blood–brain barrier by simple diffusion [33]. Thus, we measured OX-A concentrations in serum samples from LH, No-LH, and control rats to examine whether the OX-A serum concentration corresponded to OX-A neuron activation in the hypothalamus. We observed that serum OX-A concentrations in LH and No-LH rats were significantly higher than those in control rats, and the serum OX-A concentration was significantly higher in No-LH rats than in LH rats (Figure 3A). These results were consistent with the hypothalamic OX-A–positive cell counts and activation patterns. We could not detect the OX-B peptide in the serum samples. We also measured serum CORT concentrations in blood samples collected 2 h after the last AT. Both LH and No-LH rats had higher serum CORT levels than controls, and LH rats displayed significantly higher serum CORT concentrations than No-LH rats (Figure 3B).

### 3.3. OX-A and OX-B Protein Levels in Brain Areas Involved in Major Depression

**Figure 4:**
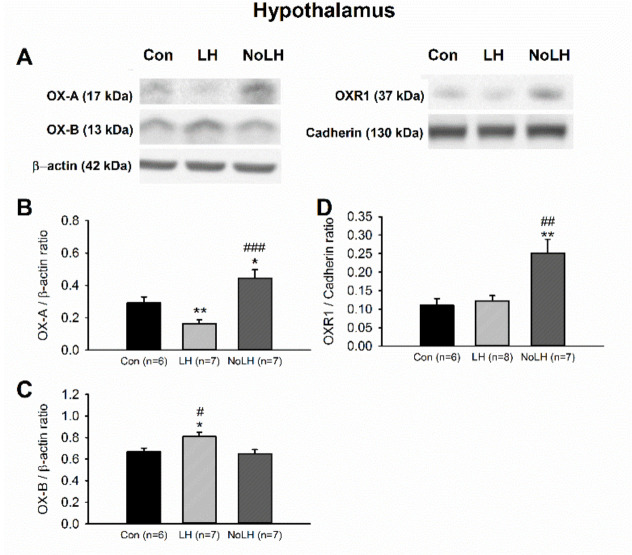
Orexin peptides and receptors in the hypothalamus of LH, No-LH, and control rats displayed different expression patterns. (**A**) Sample Western blot results. (**B**) Quantified Western blot results demonstrated that OX-A peptide was significantly reduced in LH rats, whereas No-LH rats had significantly elevated OX-A compared with LH and control rats (one-way ANOVA, between groups, *p* < 0.001; post hoc Bonferroni test, LH vs. Con, *p* = 0.036, No-LH vs. Con, *p* = 0.031, No-LH vs. LH, *p* < 0.001). (**C**) OX-B peptide was significantly elevated in LH rats (one-way ANOVA, between groups, *p* = 0.015; post hoc Bonferroni test, LH vs. Con, *p* = 0.039, No-LH vs. LH, *p* = 0.022). (**D**) OXR1 concentration was significantly increased in No-LH rats compared with LH and control rats (one-way ANOVA, between groups, *p* = 0.006; post hoc Bonferroni test, No-LH vs. Con, *p* = 0.014, No-LH vs. LH, *p* = 0.018) * *p* < 0.05, ** *p* < 0.01, compared with Con; # *p* < 0.05, ## *p* < 0.01, ### *p* < 0.001, comparison between LH and No-LH rats.

To further understand differences in the orexin system between LH and No-LH rats, we quantified the OX-A, OX-B, OXR1, and OXR2 protein levels in the brain areas involved in major depression. We first investigated orexin peptides in the hypothalamus and observed that the level of the OX-A peptide was significantly reduced in LH rats compared with that in control and No-LH rats; No-LH rats had significantly elevated OX-A levels compared with control and LH rats (Figure 4A,B). Regarding OX-B peptides, OX-B levels were significantly higher in LH rats than in No-LH and control rats (Figure 4A,C). We also observed higher expression of OXR1 in the hypothalamus of No-LH rats (Figure 4A,D). The OXR2 concentration in the hypothalamus was low and could not be reliably quantified.

**Figure 5:**
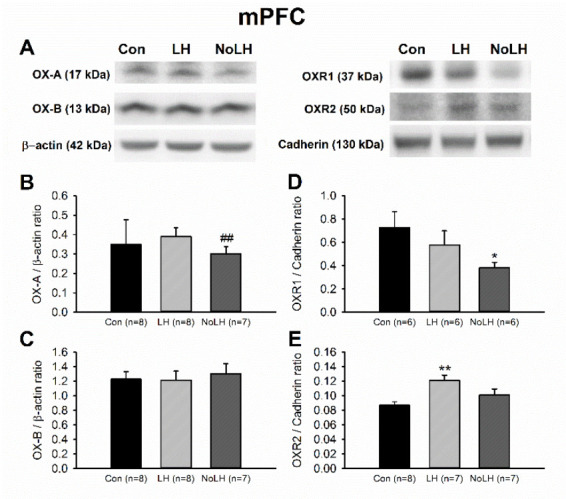
Protein levels of orexin peptides and receptors in the mPFC. (**A**) Sample Western blot results. (**B**) OX-A peptide in No-LH rats was significantly lower than that in LH rats (*t*-test, *p* = 0.002). (**C**) OX-B peptide showed no significant changes among groups. (**D**) OXR1 concentrations in No-LH rats were significantly lower than those of controls (*t*-test, *p* = 0.037). (**E**) OXR2 level was significantly elevated in LH rats compared with control rats (one-way ANOVA, between groups, *p* = 0.007; post hoc Bonferroni test, LH vs. Con, *p* = 0.005). * *p* < 0.05, ** *p* < 0.01 compared with Con; ## *p* < 0.01, comparison between LH and No-LH rats.

**Figure 6:**
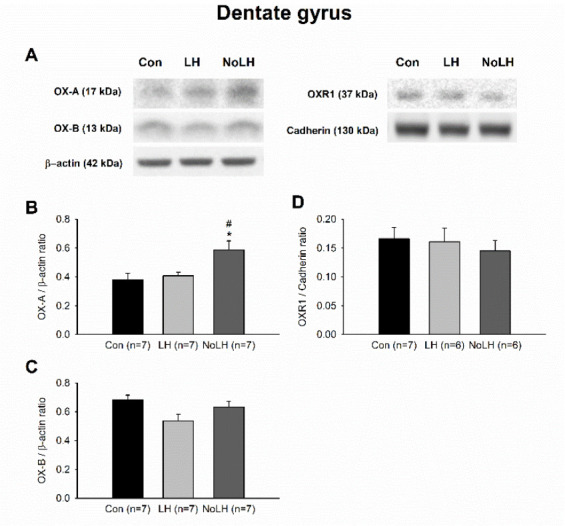
Protein levels of orexin peptides and receptors in the dorsal dentate gyrus of the hippocampal formation. (**A**) Sample Western blot results. (**B**) OX-A peptide was significantly elevated in No-LH rats compared with both LH and control groups (one-way ANOVA, between groups, *p* = 0.013; post hoc Bonferroni test, No-LH vs. Con, *p* = 0.019, No-LH vs. LH, *p* = 0.05). (**C**) OX-B peptide exhibited no significant change among groups. (**D**) OXR1 concentration was not different between groups. * *p* < 0.05, compared with Con; # *p* < 0.05, comparison between LH and No-LH rats.

**Figure 7:**
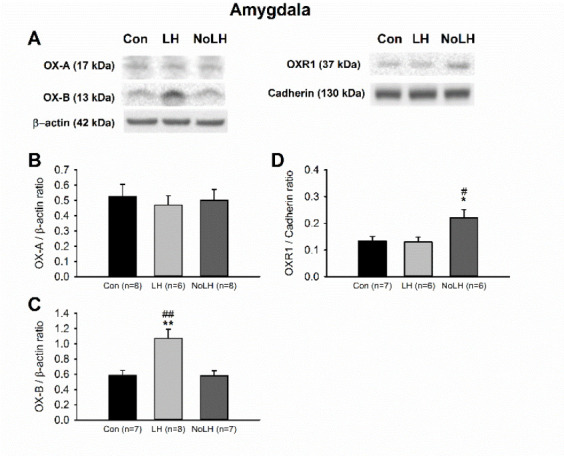
Protein levels of orexin peptides and receptors in the amygdala. (**A**) Sample Western blot results. (**B**) OX-A peptide was not different among groups. (**C**) OX-B peptide was significantly elevated in LH rats compared with both No-LH and controls (one-way ANOVA, between groups, *p* = 0.001; post hoc Bonferroni test, LH vs. Con, *p* = 0.004, LH vs. No-LH, *p* = 0.004). (**D**) OXR1 concentration was significantly elevated in No-LH rats compared with LH and controls (one-way ANOVA, between groups, *p* = 0.018; post hoc Bonferroni test, No-LH vs. Con, *p* = 0.04, No-LH vs. LH, *p* = 0.036). * *p* < 0.05, ** *p* < 0.01, compared with Con; # *p* < 0.05, ## *p* < 0.01, comparison between LH and No-LH rats.

We next examined orexin protein and receptor levels in other brain areas involved in anxiety and depression. In the mPFC, OX-A levels in No-LH rats were significantly lower than those in LH rats, and the OXR1 levels of No-LH rats were also lower than those of controls (Figure 5A,B,D). However, the OXR2 level was significantly higher in the LH group than in the control group (Figure 5A,E). In the DG of the dorsal hippocampus, we discerned a significant increase in the OX-A peptide in No-LH rats compared with LH and control rats, whereas OX-B and OXR1 levels did not differ among the groups (Figure 6). The OXR2 level in the DG was low and could not be reliably quantified. In the amygdala, the OX-A peptide concentration was not altered among the groups; however, the OX-B peptide level was elevated in LH rats compared with those in No-LH and control rats (Figure 7A–C). In addition, the ORX1 receptor level was significantly elevated in No-LH rats (Figure 7A,D). ORX2 presence in the amygdala was too low to be reliably detected.

### 3.4. OX-A and OX-B Protein Levels in Reward-Related Areas

**Figure 8:**
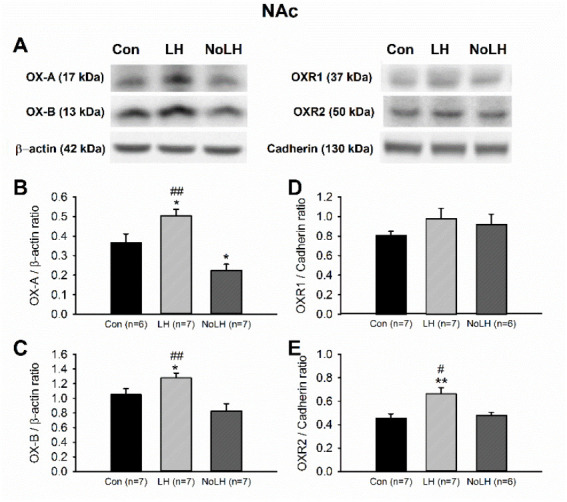
Protein levels of orexin peptides and receptors in the NAc. (**A**) Sample Western blot results. (**B**) OX-A peptide was significantly increased in LH rats compared with both No-LH and controls (one-way ANOVA, between groups, *p* < 0.001; post hoc Bonferroni test, LH vs. No-LH, *p* < 0.001, LH vs. Con, *p* = 0.016). OX-A level in the No-LH rats was significantly reduced compared with both LH and control rats (post hoc Bonferroni test, No-LH vs. Con, *p* = 0.027). (**C**) OX-B peptide was also significantly elevated in LH rats compared with both No-LH and controls (one-way ANOVA, between groups, *p* = 0.004; post hoc Bonferroni test, LH vs. No-LH, *p* = 0.003, LH vs. Con, *p* = 0.049). (**D**) OXR1 concentration was not different between groups. (**E**) OXR2 level was significantly elevated in LH rats compared with both No-LH and control rats (one-way ANOVA, between groups, *p* = 0.005; post hoc Bonferroni test, LH vs. No-LH, *p* = 0.023, LH vs. Con, *p* = 0.007). * *p* < 0.05, ** *p* < 0.01 compared with Con; # *p* < 0.05, ## *p* < 0.01 comparison between LH and No-LH rats.

**Figure 9:**
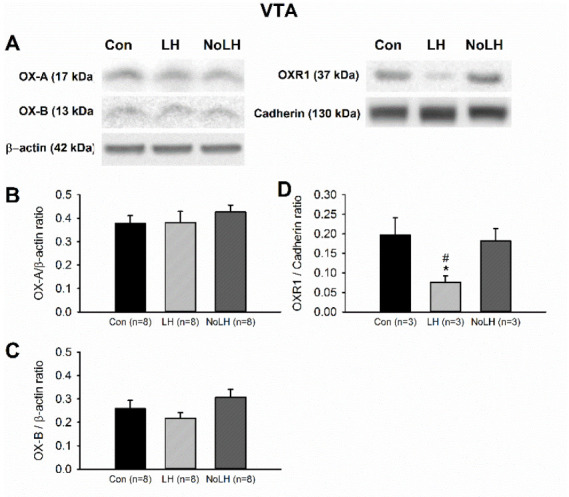
Protein levels of orexin peptides and receptors in the VTA. (**A**) Sample Western blot results. (**B**) OX-A peptide was not different among groups. (**C**) OX-B peptide was not different among groups. (**D**) OXR1 concentration was significantly reduced in LH rats compared with No-LH and controls (one-way ANOVA, between groups, *p* = 0.021; post hoc Bonferroni test, LH vs. Con, *p* = 0.033, LH vs. No-LH, *p* = 0.05). * *p* < 0.05, compared with Con; # *p* < 0.05, comparison between LH and No-LH rats.

In the NAc, we observed an increase in both OX-A and OX-B levels in LH rats compared with control and No-LH rats (Figure 8A–C). In addition, the OX-A level in the NAc of No-LH rats was significantly reduced compared with that in LH and control rats (Figure 8A,B). The OXR1 levels in the NAc did not differ among the groups, but the OXR2 concentration was significantly higher in LH rats compared with that in No-LH and control rats (Figure 8A,D,E). A prominent projection of OX neurons to the VTA was detected. In the VTA, we observed no change in either OX-A or OX-B peptide levels, but the OXR1 level was significantly reduced in LH rats (Figure 9). The OXR2 level in the VTA was too low for reliable quantification.

Overall, although the orexin cell number and activation pattern in the hypothalamus indicated that No-LH rats had more OX-A activity and that LH rats had more OX-B activity, changes in other brain areas did not always follow the same pattern.

## 4. Discussion

### 4.1. Orexin Neuronal Changes Are Associated with Depression-like Behaviors

The present study used the LH depression animal model to examine changes in the orexin system of animals displaying LH or no LH behaviors. In this model, unavoidable electrical shocks are used to induce helplessness behaviors, and ATs are used to distinguish depression-susceptible LH rats and resilient No-LH rats. Stressful electrical foot shock stimulation can activate orexin neurons in the hypothalamus [34]. We consistently found significantly increased OX-A and OX-B neuron activation in both LH and No-LH rats after shock stress. However, LH rats exhibited significantly lower OX-A–positive neuron numbers, OX-A neuron activation, OX-A concentration in the hypothalamus, and serum OX-A levels compared with the No-LH rats. These findings are in agreement with those in previous studies using the WKY genetic animal model of depression, in which the number of OX-A neurons was reduced by 18% and soma size was reduced by 15% [18]. In addition, prepro-orexin mRNA levels were reported to be reduced in male WKY rats compared with controls [17]. In another study, mice exposed to chronic mild stress also had reduced numbers of OX-A–positive neurons [35]. A social defect animal model of depression revealed a reduction of hypothalamic OX-A and OX-B levels in rats displaying anhedonic behaviors [36]. Thus, the reduction of OX-A neurons and their activation may be associated with depression-like behaviors. In addition, studies using the dual orexin receptor antagonist almorexant have implied that orexins are involved in stress-induced arousal, particularly when it is associated with responses that require increased attention to environmental cues [34]. Although No-LH rats were less stressed than LH rats in this study, they had higher numbers and activation of OX-A neurons, suggesting that the activation of hypothalamic OX-A neurons might contribute to stress resilience or a more favorable adaptive response to stress, whereas a reduction in OX-A neurons could cause rats to become more vulnerable to stressful stimuli and to exhibit depression-like behaviors similar to those observed in WKY rats and LH rats. Using a psychosocial stress model, a recent study also demonstrated that orexin prevented depression-like behaviors by promoting stress resilience in stressed rats [37]. By contrast, in another genetic depression model, female FSL rats that demonstrated depression-like behaviors had more OX-A neurons than the control strain of Flinders Resistant Line [19]. The causes of the discrepancies between FSL rats and WKY rats regarding OX-A neuronal changes are unclear. Sex differences might be a factor, but another possibility is that the increased number of OX-A neurons in FSL rats is a compensatory effect. In addition to depression-like behaviors, FSL rats display sleep dysfunction, appetite problems, and hypersensitivity to stress, whereas WKY rats display only sleep dysfunction [17,19]. Based on the findings of the aforementioned studies and our results, we suggest that the OX-A system facilitates appropriate adaptive behaviors promoting stress resilience and a reduction in depression-like behaviors. In addition to increased OX-A neuron activation in the No-LH rats, we observed an increase in the OX-B neuron activity in the LH rats. Since OX-B has a higher affinity for OXR2 than OXR1, there might be more activation of the OXR2 in LH rats. Although a majority of the anxiety and depression studies focused on OXR1, there was accumulating evidence suggesting OXR2 activity is anxiolytic [24,38,39]. Mice susceptible to anxious and depressive behavior in a social defeat model displayed reduced OXR2 mRNA, this reduction is not observed in resilient mice [24]. We speculate that the increased OX-B neuron activation in LH rats is a compensatory response to boost the OXR2 activation. However, this hypothesis will require further research to substantiate. Furthermore, since OX-A and OX-B are produced by the same precursor protein, there may have been a tendency to assume that these two peptides are produced equally in each orexin neuron. However, there is evidence that the up-regulation of one orexin peptide over the other is possible [40,41]. There might be a small subpopulation of orexin neurons in which these two orexin peptides are differentially regulated for specific function.

### 4.2. Orexin Changes in PVN

Studies have shown that intraventricular injection of OX-A can increase the activation of the paraventricular nucleus of the hypothalamus (PVN), elevate the CRH level in the PVN, and increase both ACTH and CORT in the blood [42,43]. The PVN is crucial in controlling the hypothalamic-pituitary-adrenal (HPA) axis and has abundant OX-A immune-reactive fiber projections [1]. Although we observed more OX-A activity in No-LH rats in this study, LH rats had higher serum CORT levels. Therefore, the enhanced CORT response in LH rats is unlikely to be mediated solely by OX-A stimulation. In addition, our previous studies have demonstrated that the serum CORT level of No-LH rats that received the same number of electrical shocks as LH rats were still significantly lower than that of LH rats; therefore, the higher CORT level in LH animals cannot be simply attributed to their having received more electrical shocks than No-LH rats [44]. Alternatively, the elevated CORT in LH rats might be caused by impaired HPA axis feedback regulation; in the prenatal polysaccharide-exposure animal model of depression, the reduction in hippocampal glucocorticoid receptor levels might contribute to the dysregulation of the HPA axis [45]. Furthermore, studies using optogenetic tools have demonstrated distinct subpopulations of CRF- and cholecystokinin (Cck)-containing neurons in the bed nucleus of stria terminalis (BNST) project to different populations of orexin neurons in the lateral hypothalamus; and display discrete responses to aversive and rewarding stimuli [46]. Thus, the BNST neurons may also play a role in regulating the orexin system in stressful conditions.

### 4.3. Orexin Changes in Brain Areas Associated with Processing Fearful Memories

Orexin neurons have projections throughout the brain, including the prefrontal cortex and several limbic areas [47]. In the prelimbic area of the mPFC, LH rats had significantly higher OXR2 levels than controls. However, No-LH rats tended to have lower OX-A levels and fewer OXR1 receptors. Orexins are considered excitatory neuropeptides, as OXRs are coupled to effectors that depolarize the neurons [48]. Because animal studies have suggested that the prelimbic area is involved in fear expression and aversive memories [49], the higher excited state of the prelimbic area in LH rats might facilitate the development of fear-related behaviors and the formation of aversive memory, which could contribute to LH behaviors in rats. The hippocampus and amygdala are also involved in emotional memory consolidation and extinction [50,51]. In a previous study, the FST in mice demonstrated that the interventricular injection of OX-A had an antidepressant effect; it facilitated hippocampal neurogenesis and reduced immobility [20]. Furthermore, the OX-A concentration in the dorsal hippocampus was shown to be negatively correlated with the immobility time in the FST, suggesting that a higher concentration of OX-A in the hippocampus is associated with lower depressive behavior [23]. No-LH rats had significantly elevated OX-A in the hippocampus. The increase in OX-A may facilitate neurogenesis and reduce depression-like behaviors in No-LH rats. We did not examine hippocampal neurogenesis in the present study; however, our previous studies have demonstrated that LH rats have reduced neurogenesis compared with No-LH rats [44]. The relationship between increased OX-A and hippocampal neurogenesis in No-LH rats warrants further study.

The amygdala is crucial for the acquisition and extinction of fear memory [47,52,53]. Amygdala activation promotes anxiety and stress responsiveness and is related to the depression phenotype [23,54]. Based on FST results, studies have reported a U-shaped relationship between the immobility time and OX-A in the amygdala and a weak positive correlation between the immobility time and OXR1 gene expression, suggesting that OXR1 in the amygdala is associated with behavioral despair [23]. Furthermore, in a social defeat chronic stress model of mice, the OXR1 level was increased, and the OXR2 level was decreased in the amygdala of mice susceptible to chronic defeat; therefore, OXR1 is considered to be anxiogenic, whereas OXR2 is anxiolytic [24]. In the current study, to our surprise, we observed more OXR1 in the amygdala of No-LH rats than in LH rats. OXR1 in the amygdala might increase anxiety in No-LH rats, and the enhanced vigilance might be beneficial for coping with stressful situations. In addition, in this study, we discerned increased OX-B levels in the amygdala of LH rats. Because the binding affinity of OX-B to OXR1 is much lower than that of OX-A [48], increased OX-B levels could have more effects on the functions mediated by OXR2 than on those mediated by OXR1. Given that studies have indicated that the activation of OXR2 in the basolateral amygdala (BLA) is involved in anxiolytic functions [24], the increase in OX-B in LH rats is possibly an adaptive response to reduce shock stress. Excessive activation of OXR2 in the BLA might render LH rats into a state more submissive to stress. In addition, because two populations of BLA neurons encode fear conditioning and extinction processes [53], the location of OXRs would determine their functional significance.

### 4.4. Orexin Changes in Brain Areas Involved in Reward Processing

Anhedonia is a major symptom of depression. The patient loses interest in pleasurable things that would normally be considered rewarding. Clinical studies have indicated that the NAc may be responsible for anhedonia in major depression because unmedicated patients with depression exhibited reduced NAc activity in response to rewards [55]. Among reward-related brain areas, we examined the VTA and the NAc in this study. Orexin has been shown to increase the excitatory postsynaptic current in dopaminergic neurons in the VTA [56,57], and direct microinjection of OX-A or OX-B in the VTA increased dopamine concentrations in the NAc and reward seeking behaviors [58,59]. We observed that OXR1 was significantly reduced in the VTA of LH rats. The reduction in OXR1 in LH rats might reduce the excitation of dopaminergic neurons in the VTA and consequently reduce dopamine release in the NAc. As a result, the activation of the reward pathway may be decreased in LH rats, and the reduced dopaminergic activity in the NAc may also affect anhedonic behaviors. In addition to reward-related behaviors, the NAc has been shown to be involved in feeding and fear; in particular, orexin receptors in the NAc are involved in regulating stress-induced drug reinstatement [60,61,62]. In the NAc, we noted a significant increase in OX-A, OX-B, and OXR2 levels in LH rats in this study, whereas OX-A levels were significantly reduced in No-LH rats8). Because OX-A and OX-B play excitatory roles in neurotransmission, increased OX-A and OX-B production in LH rats might increase glutamatergic transmission in the NAc. Studies have determined that the blockade of glutamatergic excitation in the NAc shell might increase feeding and defensive behaviors in rats [61]; therefore, enhanced glutamatergic transmission in LH rats could reduce their feeding and defensive behaviors, which is in agreement with a depression-like behavioral phenotype. However, the circuitry and connection of the NAc are complex, and the orexin system in this area also interacts with dopaminergic signals from the VTA; therefore, the functional role of orexins in integrating stress-related responses require further research.

## 5. Conclusions

The location and wide projection of the hypothalamic orexin system make it a possible candidate with multiple roles in regulating physiological and emotional functions. The current study found various changes in orexin peptides and receptors in multiple brain areas involved in stress and depressive behaviors. Although the neuronal circuits underlying the behavioral differences between LH and no LH are complicated, we hope that by observing local changes of the orexin system in the brain areas involved in major depression, we can gain more insights into the pathophysiological changes underlying depression-like behaviors.

## Data Availability

Data is contained within the article or supplementary material.

## Supplementary Materials

The following are available online at www.mdpi.com/article/10.3390/brainsci11121634/s1, Figure S1: Shock time of the second AT; Figure S2: Brain tissue sampling location.
