# Supplementary material for: Changes in the Orexin System in Rats Exhibiting Learned Helplessness Behaviors
Source: Brain Sci. 2021 Dec 10;11(12):1634. doi: 10.3390/brainsci11121634 (PMC8699801; doi:10.3390/brainsci11121634)
Supplement: Supplementary file 1 [file brainsci-11-01634-s001.zip › brainsci-1448565-supplementary.pdf]

Supplemental material

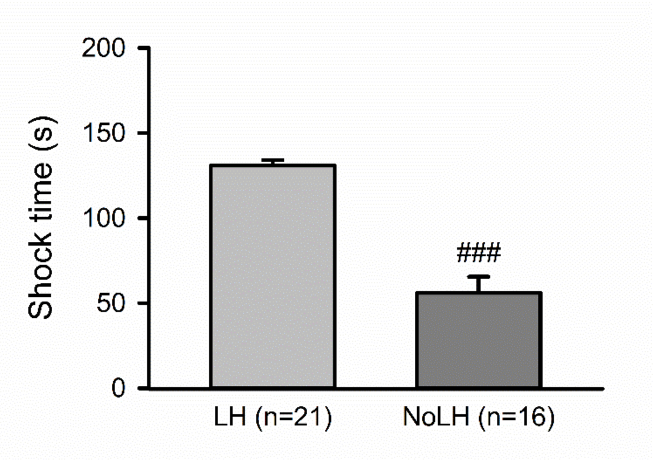


**Figure S1.** Shock time of the second AT. On average, the LH rats was shocked 130.9 ± 3.2 s whereas No-LH rat was shocked 56 ± 9.5 s. At the first AT, there were 22 LH rats and 15 No-LH rats. At the second AT, one of the LH rats from the first AT became No-LH rats. All the No-LH rats from the first AT test remained to be No-LH rats at the second AT.

(A)

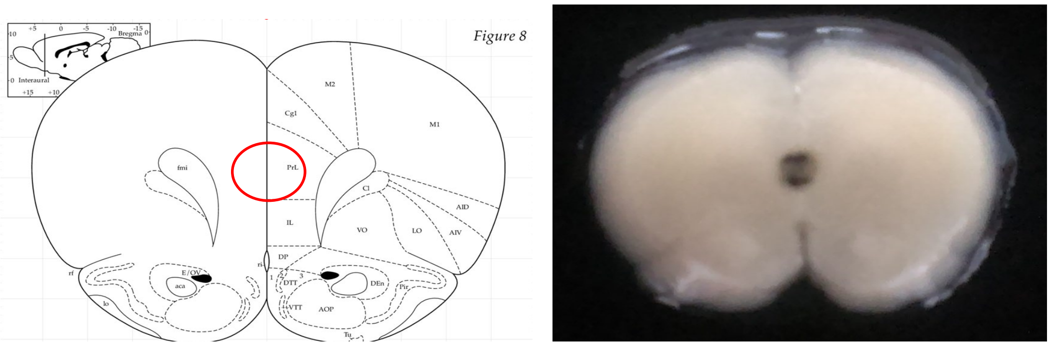


(B)

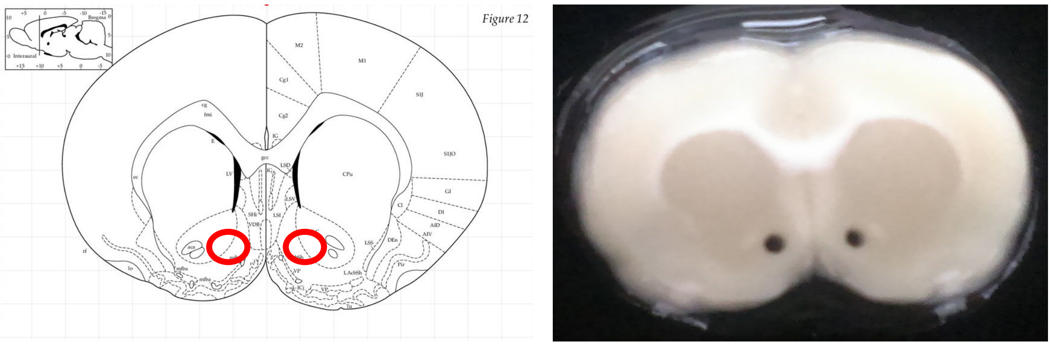


(C)

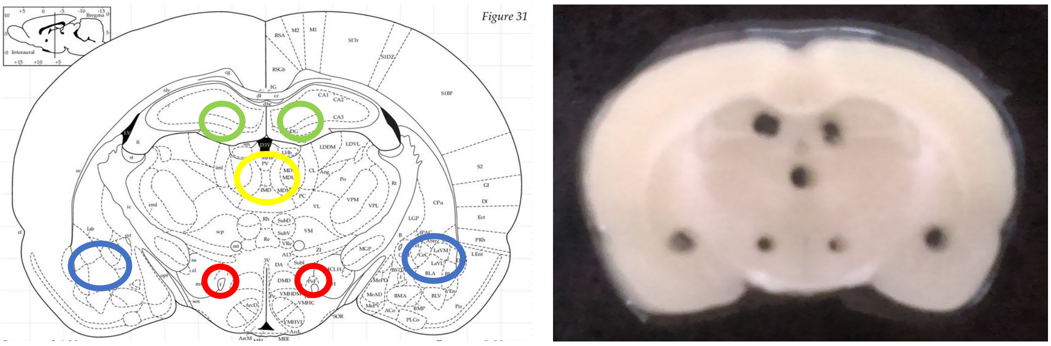


(D)

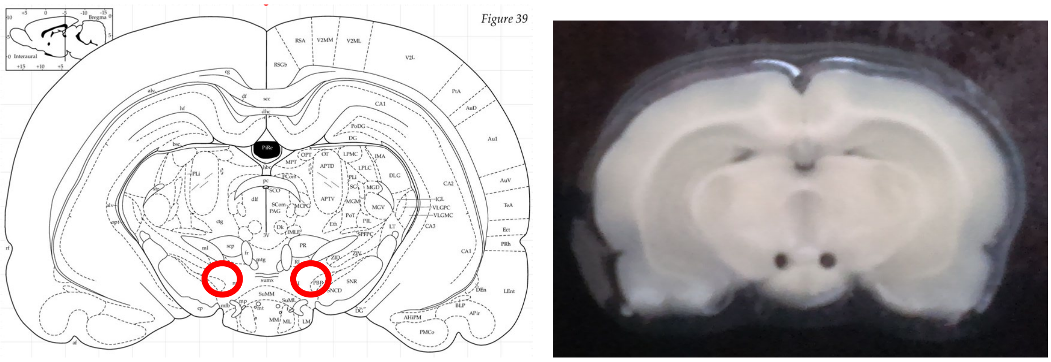


**Figure S2**. Brain tissue sampling location

(A) Prelimbic medial prefrontal cortex prelimbic area (B) Nucleus accumbens, including parts of core and shell area (C) Dentate gyrus of hippocampus (green); Paraventricular nucleus of thalamus (yellow); Amygdala (blue); Hypothalamus (red) (D) Ventral tegmental area
